# Risk systems: identifying the key components and interdependencies of a mental health system in which risk decisions are made

**DOI:** 10.1192/j.eurpsy.2026.11280

**Published:** 2026-07-31

**Authors:** R. Nathan, S. Jones, A. Brown

**Affiliations:** 1 University of Chester; 2 Cheshire and Wirral Partnership NHSFT, Chester; 3 Mersey Care NHSFT, Liverpool, United Kingdom

## Abstract

**Introduction:**

A consensus has yet to be reached on whether the appraisal of risk is best represented quantitatively (e.g. probability estimates) or qualitatively (e.g. formulation). A more fundamental issue, though, is how to use the agreed representation to inform practice so as to reduce the likelihood of harm. The real-world implementation of risk representations in clinical practice is dependent on how these representations inform clinical decision-making. Clinical decision-making takes place in a complex system of interacting agents, entities, and processes. To date, risk-related research and practice guidance has not paid sufficient attention to studying this system.

**Objectives:**

To develop a standardised model for representing those complex mental health systems in which appraisals of ‘risk of harm to others’ are used.

**Methods:**

In order to identify a generally applicable representation of the system, practice experts in the field of risk of harm to others reflexively examined the systems within which they worked to firstly identify the actual individual agents, entities and processes in their systems. A thematic review of the elements was then undertaken so as the inform the development of more generally applicable characterisation of the system for wider use.

**Results:**

The generalisable characterisation of the system (with illustrative local examples in parenthesis) is presented in Figure 1.

**Image 1:**
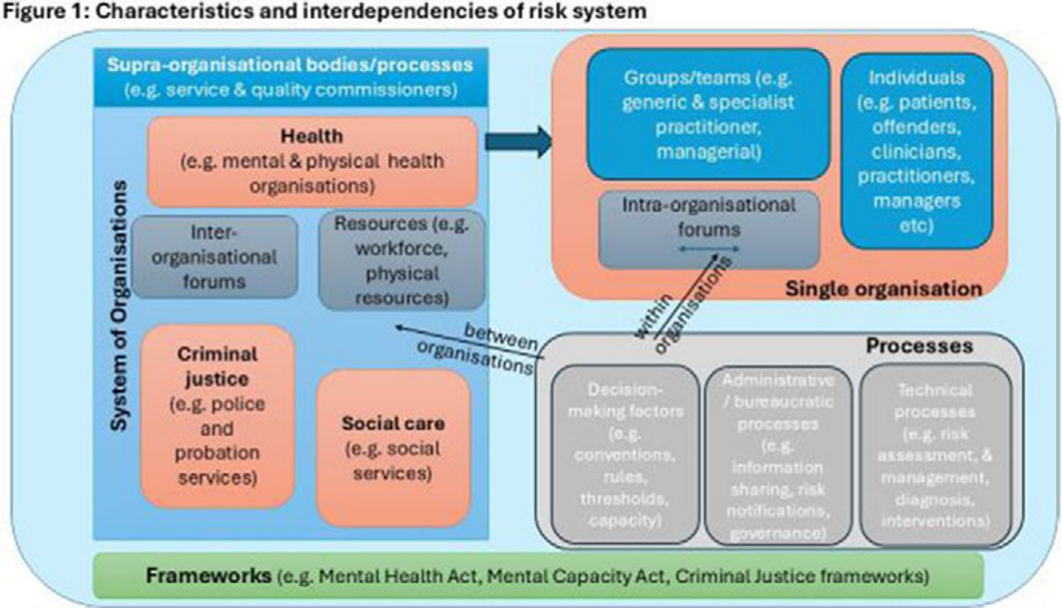
Image 1: Long description.

**Conclusions:**

Risk-related practice by mental health clinicians involves the development of quantitative and/or qualitative representations of risk which are used to guide decisions. In practice, the use of the risk representations to guide decisions is also heavily dependent on the complex system in which the decisions are made. This study has generated a model to characterise risk-related systems which can be used for service improvement and planning, and for future research into risk-related practice.

**Disclosure of Interest:**

None Declared

## Long descriptions

### Image 1: Long description

The flowchart is divided into three primary zones.

1. System of Organisations (Left): A large blue rectangle labeled Supra-organisational bodies/processes contains four sub-boxes. Health (mental and physical health organisations) and Criminal justice (police and probation services) are in orange. Inter-organisational forums and Resources (workforce, physical resources) are in grey. A vertical label on the left reads System of Organisations. An arrow points from the Health box toward the Single organisation zone.

2. Single Organisation (Top Right): An orange rectangle containing three blue and grey boxes. Groups/teams (generic and specialist practitioner, managerial) and Individuals (patients, offenders, clinicians, practitioners, managers etc) are in blue. Intra-organisational forums is in grey. A double-headed arrow labeled ‘within organisations’ connects this zone to the Processes zone.

3. Processes (Bottom Right): A grey rectangle containing three sub-categories: Decision-making factors (conventions, rules, thresholds, capacity), Administrative / bureaucratic processes (information sharing, risk notifications, governance), and Technical processes (risk assessment, management, diagnosis, interventions). A double-headed arrow labeled ‘between organisations’ connects this zone back to the System of Organisations.

4. Frameworks (Bottom): A green horizontal bar spanning the width of the diagram labeled Frameworks (e.g. Mental Health Act, Mental Capacity Act, Criminal Justice frameworks).

Navigate back to Image 1:.

